# Metagenomes of Wastewater at Different Treatment Stages in Central Germany

**DOI:** 10.1128/MRA.00201-20

**Published:** 2020-04-09

**Authors:** Dominik Schneider, Nils Aßmann, Dennis Wicke, Anja Poehlein, Rolf Daniel

**Affiliations:** a Genomic and Applied Microbiology and Göttingen Genomics Laboratory, Institute of Microbiology and Genetics, Georg-August University of Göttingen, Göttingen, Germany; b Microbiology and Biochemistry MSc/PhD Program, Georg-August University of Göttingen, Göttingen, Germany; Georgia Institute of Technology

## Abstract

Nine metagenomes derived from university hospital effluent, at different stages of wastewater treatment, and the river adjacent to the wastewater treatment plant in Göttingen, Germany, were analyzed. *Bacteria* was the dominant domain and mainly comprised *Proteobacteria*, *Firmicutes*, *Bacteroidetes*, and *Actinobacteria*. The microbiomes harbored a diverse microbial community with a site-specific structure.

## ANNOUNCEMENT

The metagenomics of wastewater samples provides important insights with respect to human health-related factors (1), including distribution of pathogens and antibiotic resistance genes.

**Fig 1:**
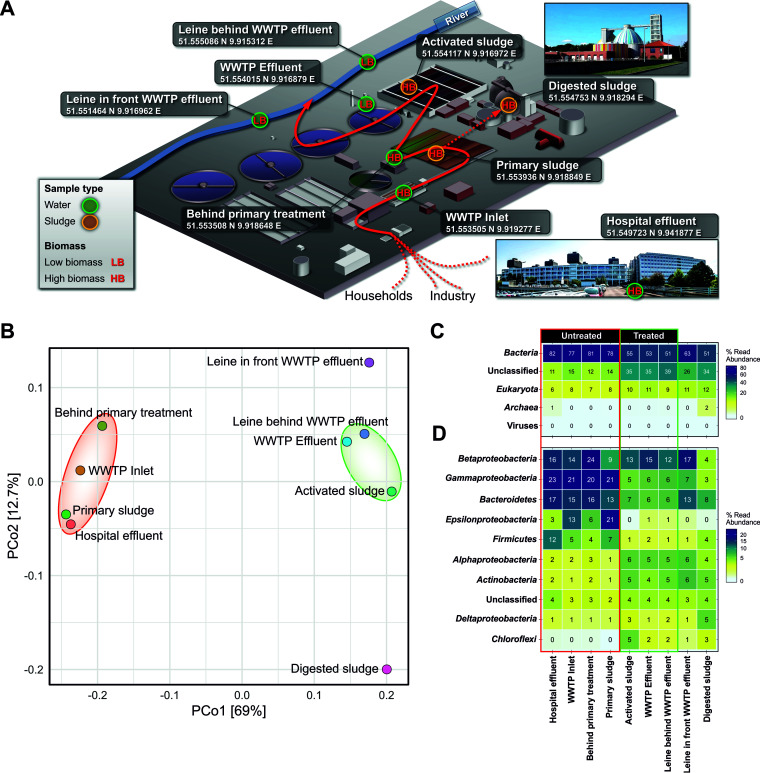
Sample locations and taxonomic composition of the microbial community isolated from different stages of wastewater treatment. (A) Scheme of the WWTP in Göttingen, Germany, and the sampling sites, including latitude and longitude. (B) Principal coordinate analysis (PCoA)-based taxonomic composition calculated with Bray-Curtis (13) distance matrices. (C) Domain composition. (D) The 10 most abundant bacterial phyla; however, *Proteobacteria* are shown at the class level. Untreated and treated wastewater samples are highlighted in panel B by red and green circles, respectively, and in panels C and D by red and green boxes, respectively.

Municipal wastewater, hospital wastewater, sludge, and river samples of nine different locations were collected in the area of the wastewater treatment plant (WWTP) of Göttingen, Germany, in November 2016. The samples (three technical replicates each) were collected from a storage tank next to the university hospital (hospital effluent), a WWTP inlet, a spot directly after the primary treatment unit, primary sludge, activated sludge, digested sludge, WWTP effluent, and the Leine River in front of and behind the WWTP discharge (Fig. 1A). Water samples of the WWTP were collected using a collection device which collects water for 24 h according to the flowthrough rate; all other samples were collected manually. Subsequently, the planktonic fraction was harvested by centrifugation (15 min at 11,000 × *g* and 4°C). The recovered pellets were mixed in a 1:1 ratio with RNAprotect (Qiagen, Hilden, Germany) and stored at 4°C. For DNA isolation, RNAprotect was removed by centrifugation, and DNA was extracted using the PowerSoil DNA isolation kit (Mo Bio Laboratories, Inc., Carlsbad, CA). The DNA isolations of each sampling site replicate were pooled equimolar. The sequencing libraries were constructed and indexed with a Nextera DNA sample preparation kit and index kit as recommended by the manufacturer (Illumina, San Diego, CA, USA). Paired-end sequencing was performed using a HiSeq 2500 instrument (rapid run mode, 500 cycles) as recommended by the manufacturer (Illumina). Data processing included fastp v0.20.0 (2) with overlap correction, quality filtering (removal of reads with a quality score of <Q20 and clipping with a sliding window setting of 4), and adapter removal. Default parameters were used for all software unless otherwise specified. After quality filtering, the metagenomes consisted of 350 million paired-end reads and ranged from 34.7 million to 45.8 million reads per sample (average, 39 million reads per sample). An average read length of 199 bp was recorded for the forward reads, and an average of 198 bp was recorded for the reverse reads. Taxonomic classification of the paired-end reads was performed using a combination of Kraken 2 (3) and Kaiju (4) against the NCBI nucleotide nonredundant (nt/nr) database (downloaded on 18 January 2020), but the Kraken 2 classification was favored over the Kaiju classification (5). Analysis and visualization were performed with R (6), RStudio (7), and ampvis2 (8).

Principal coordinate analysis (PCoA) based on the taxonomic composition showed that untreated wastewater and samples collected during and after treatment formed distinct clusters (Fig. 1B). The separation of the digested sludge sample indicated a specific microbial community, which is most likely shaped by the anaerobic conditions in the digestion tower. From all reads, 50.9 to 81.7% were classified as bacteria, 0.2 to 2.5% as archaea, 0.1 to 1.4% as eukaryotes, and 0.2 to 0.3% as viruses. In addition, 11.3 to 38.8% of the reads could not be classified (Fig. 1C). A shift in bacterial composition was recorded during the transition from untreated to processed wastewater. The most prominent phyla were *Proteobacteria*, *Bacteroidetes*, and *Firmicutes* (Fig. 1D). The microbial community composition detected at the phylum level is in line with that in previous studies targeting microbial communities of WWTPs (9–12).

### Data availability.

These metagenome sequences have been deposited in the NCBI Sequence Read Archive under the accession numbers SRX5445792 (Leine River after WWTP), SRX5445793 (Leine River before WWTP), SRX5445794 (WWTP inlet), SRX5445795 (WWTP effluent), SRX5445796 (primary sludge), SRX5445797 (digested sludge), SRX5445798 (activated sludge), SRX5445799 (WWTP after primary treatment), and SRX5445800 (university hospital effluent). The BioProject number is PRJNA524094.
